# New Synthesis, Structure and Analgesic Properties of Methyl 1-R-4-Methyl-2,2-Dioxo-1*H*-2λ^6^,1-Benzothiazine-3-Carboxylates

**DOI:** 10.3390/scipharm85010002

**Published:** 2017-01-12

**Authors:** Liliana Azotla-Cruz, Irina V. Lijanova, Igor V. Ukrainets, Natalya V. Likhanova, Octavio Olivares-Xometl, Natalya L. Bereznyakova

**Affiliations:** 1 Department of Investigation and Postgrad, National Polytechnic Institute, CIITEC, Cerrada Cecati S/N, Colonia Santa Catarina de Azcapotzalco, CP 02250 México, DF, Mexico; lilianazotla@hotmail.com (L.A.-C.); irinalijanova@yahoo.com.mx (I.V.L.); 2 Department of Pharmaceutical Chemistry, National University of Pharmacy, 53 Pushkinska St., 61002 Kharkiv, Ukraine; talaber@mail.ru; 3 Management of Additional Oil Recovery Engineering, Mexican Institute of Petroleum, Eje Central Lázaro Cárdenas Norte 152, Col. San Bartolo Atepehuacán, 07730 México, DF, Mexico; nvictoro@imp.mx; 4 Department of Engineering Chemistry, Meritorious Autonomous University of Puebla, Chemical Engineering, Av. San Claudio, Ciudad Universitaria, Col. San Manuel, 72570 Puebla, Pue., Mexico; oxoctavio@yahoo.com.mx

**Keywords:** alkyl 4-methyl-2,2-dioxo-1*H*-2λ^6^,1-benzothiazine-3-carboxylate, alkylation, analgesic activity, 2,1-benzothiazine, bioisosteric replacements, crystal structure

## Abstract

According to the principles of the methodology of bioisosteric replacements a series of methyl 1-R-4-methyl-2,2-dioxo-1*H*-2λ^6^,1-benzothiazine-3-carboxylates has been obtained as potential analgesics. In addition, a fundamentally new strategy for the synthesis of compounds of this chemical class involving the introduction of *N*-alkyl substituent at the final stage in 2,1-benzothiazine nucleus already formed has been proposed. Using nuclear magnetic resonance (NMR) spectroscopy, mass spectrometry and X-ray diffraction analysis it has been proven that in the DMSO/K_2_CO_3_ system the reaction of methyl 4-methyl-2,2-dioxo-1*H*-2λ^6^,1-benzothiazine-3-carboxylate and alkyl halides leads to formation of *N*-substituted derivatives with good yields regardless of the structure of the alkylating agent. The peculiarities of NMR (^1^Н and ^13^С) spectra of the compounds synthesized, their mass spectrometric behavior and the spatial structure are discussed. In *N*-benzyl derivative the ability to form a monosolvate with methanol has been found. According to the results of the pharmacological testing conducted on the model of the thermal tail-flick it has been determined that replacement of 4-ОН-group in methyl 1-R-4-hydroxy-2,2-dioxo-1*H*-2λ^6^,1-benzothiazine-3-carboxylates for the methyl group is actually bioisosteric since all methyl 1-R-4-methyl-2,2-dioxo-1*H*-2λ^6^,1-benzothiazine-3-carboxylates synthesized demonstrated a statistically significant analgesic effect. The majority of the substances can inhibit the thermal pain response much more effective than piroxicam in the same dose. Under the same conditions as an analgesic the *N-*methyl-substituted analog exceeds not only piroxicam, but more active meloxicam as well. Therefore, it deserves in-depth biological studies on other experimental models.

## 1. Introduction

The topical issues of pharmaceutical and medical chemistry are the search of lead compounds with the high analgesic activity, their subsequent optimization and creation of new pain killers, which can meet the current requirements for efficiency and safety of medicines. The concept of bioisosteric replacements has proven to be one of the successful and fruitful methods for solving problems of this type [1,2]. By now it has become a powerful tool for purposeful synthesis of biologically active substances not only with the desired pharmacological activity, but with improved characteristics compared to the structures-prototypes [3,4,5,6,7,8,9].

**Figure 1:**
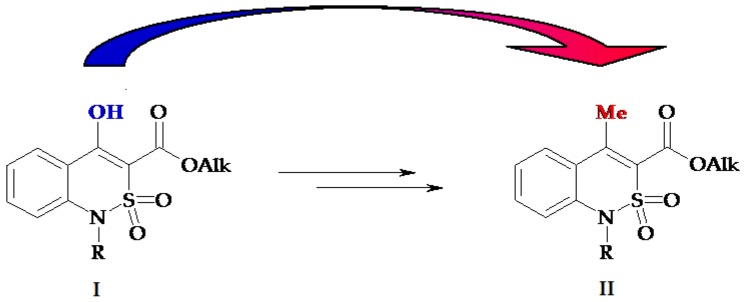
The potentially bioisosteric replacement [1,2] from methyl 1-R-4-hydroxy-2,2-dioxo-1*H*-2λ^6^,1-benzothiazine-3-carboxylates **I** to their 4-methyl-substituted analogs **II**.

The interesting objects of study in this respect are various derivatives 4-hydroxy-2,2-dioxo-1*H*-2λ^6^,1-benzothiazine-3-carboxylic acids. We have chosen these compounds for a number of reasons. First, because of synthetic difficulties, they still remain chemically and pharmacologically understudied, and it makes them particularly attractive from the point of view of novelty that is important for any innovation project. Secondly, having synthesized and studied only approximately 200 compounds of this chemical class we succeeded in demonstrating convincingly that esters (especially methyl esters) [10,11], arylalkylamides [12,13,14], anilides [15,16,17] and hetarylamides [18,19] of 1-R-4-hydroxy-2,2-dioxo-1*H*-2λ^6^,1-benzothiazine-3-carboxylic acids have real prospects for developing highly effective analgesics. Finally, another important factor is that derivatives of 4-hydroxy-2,2-dioxo-1*H*-2λ^6^,1-benzothiazine-3-carboxylic acids and, first of all, their esters of the general formula **I** (Figure 1), possess a wide synthetic potential allowing to make a variety of chemical transformations to the basic molecule virtually unrestrictedly and thereby to change its pharmaceutical and (or) pharmacological properties in the right direction. 

Even a cursory analysis of the structure of esters **I** shows that here the replacement of 4-ОН-group by one of its classic isosteres suggests itself as a most obvious variant of the methodology of bioisosteric replacements [1,2], for example by the methyl group. However, only experimental pharmacological testing will show whether transfer from 4-ОН-esters **I** to their 4-methyl-substituted analogs **II** (Figure 1) will be really bioisosteric and how exactly this transformation will affect the analgesic properties of the compounds under study.

**Scheme 1:**
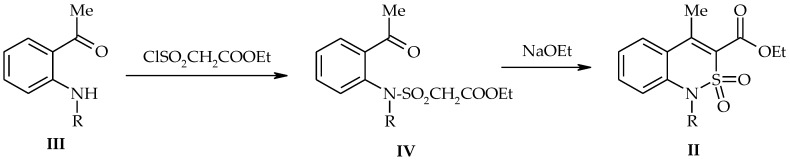
Known method for the synthesis of ethyl 1-R-4-methyl-2,2-dioxo-1*H*-2λ^6^,1-benzothiazine-3-carboxylates **II** [22,23].

It may happen that the above transformation of the ОН-group into the methyl one can be performed via the intermediate formation of the corresponding 4-Cl-derivatives with their subsequent treatment with methylene active compounds followed by alkaline hydrolysis. At least, in the case of structurally similar alkyl 1-R-4-hydroxy-2-oxo-1,2-dihydroquinoline-3-carboxylates this method has shown excellent results [20,21]. Nevertheless, a completely different principle of formation of molecular systems of type **II** is known (Scheme 1). It is acylation of *ortho*-aminoacetophenones **III** with ethyl (chlorosulfonyl)acetate, after that *ortho*-acetylsulfonamides **IV** formed are subjected to intramolecular cyclization in the target benzothiazines **II** under the influence of bases [22,23].

Undoubtedly, the weakest link in this synthetic chain is the use of *N*-substituted *ortho*-aminoacetophenones **III**. Basically, this approach is entirely justified when obtaining esters **II** (R = H) that are unsubstituted in position 1, in their synthesis the use of commercially available acetophenones **III** with a free amino group is expected. However, introduction of *N*-alkyl substituents directly to the molecule of *ortho*-aminoacetophenone is associated with rather serious difficulties conditioned by both its extreme chemical instability and the secondary processes that are inevitable for such reactions (formation of *N*,*N*-dialkylation products, etc.). Hence, it is not surprising to make attempts to obtain 1-substituted esters **II** (R = alkyl) in some other manner that excludes the need for alkylation of *ortho*-aminoacetophenones. Although, in general, there are no complaints to the sequence of chemical transformations proposed in the method under consideration since the time-tested strategic rule of organic synthesis recommending all controversial and difficult stages to transfer to the beginning of the synthetic scheme has been fully complied [24]. In light of this, the possibility of obtaining 1-alkyl-substituted esters **II** by the fundamentally different way has been studied in the present work when the desired *N*-alkyl substituent is not introduced at first stage, but it is introduced at the final stage of the synthesis, i.e., by alkylation of the 4-methyl-2,2-dioxo-1*H*-2λ^6^,1-benzothiazine nucleus previously formed.

## 2. Materials and Methods

### 2.1. Chemistry

^1^Н- and ^13^С-NMR spectra were acquired on a Varian Mercury-400 (Varian Inc., Palo Alto, CA, USA) instrument (400 and 100 MHz, respectively) in dimethyl sulfoxide-*d*_6_ (DMSO-*d*_6_), chloroform-*d* (CDCl_3_) or their mixtures in the ratio of 1:1 with tetramethylsilane as internal standard. The chemical shift values were recorded on a δ scale and the coupling constants (*J*) in hertz. The following abbreviations were used in reporting spectra: s = singlet, d = doublet, t = triplet, q = quartet, m = multiplet. The electron impact mass spectra were recorded on a Varian 1200 L (Varian Inc.) mass spectrometer with complete scanning in the *m*/*z* range from 35 to 700 and direct sample inlet. The electron impact ionization was at 70 eV. Elemental analysis was performed on a Euro Vector EA-3000 (Eurovector SPA, Redavalle, Italy) microanalyzer. Melting points were determined in a capillary using a Electrothermal IA9100X1 (Bibby Scientific Limited, Stone, UK) digital melting point apparatus. Monitoring of alkylation and purity of the compounds obtained was done by thin-layer chromatography (TLC) on Silica gel 60 F_254_ aluminium sheets (Merck KGaA, Darmstadt, Germany); the eluent was CH_2_Cl_2_-MeOH-hexane, 5:1:5; visualization of the chromatograms was with iodine vapor. In the synthesis of methyl 1-R-4-methyl-2,2-dioxo-1*H*-2λ^6^,1-benzothiazine-3-carboxylates **3** and **5** described in this article the commercial *ortho*-aminoacetophenone and the corresponding alkyl halides of Sigma Aldrich company (St. Louis, MO, USA) were used.

### 2.2. Methyl 4-Methyl-2,2-Dioxo-1*H*-2λ^6^,1-Benzothiazine-3-Carboxylate (**3**)

Add methyl (chlorosulfonyl)acetate (1.90 g, 0.011 mol) dropwise with stirring to the solution of *ortho*-aminoacetophenone (**1**) (1.35 g, 0.010 mol) and triethylamine (1.54 mL, 0.011 mol) in CH_2_Cl_2_ (20 mL) and cool (−5 to 0 °C). After 10 h add water (50 mL) to the reaction mixture, then acidify it to pH 4 with 1 N HCl and mix thoroughly. Separate the organic layer, dry over anhydrous CaCl_2_, and distil the solvent (at reduced pressure at the end). Subject the resulting anilide **2** to heterocyclization without purification. Add the solution of sodium methylate in anhydrous methanol (from metallic sodium (0.69 g, 0.030 mol) and absolute methanol (15 mL)), boil the mixture and store for 15 h at room temperature. Dilute the reaction mixture with cold water and acidify with 1 N HCl to pH 4. Filter the solid ester **3** separated, wash with water, and dry in the air. Yield: 2.25 g (89%); colorless crystals; melting point (mp) 203–205 °C (methanol); retention factor (R*_f_*) 0.37; ^1^H-NMR (400 MHz, DMSO-*d*_6_): δ 11.84 (br. s, 1H, NН), 7.79 (d, 1Н, *J* = 7.6 Hz, Н-5), 7.49 (t, 1Н, *J* = 7.2 Hz, Н-7), 7.22 (t, 1Н, *J* = 7.6 Hz, Н-6), 7.12 (d, 1Н, *J* = 8.0 Hz, Н-8), 3.84 (s, 3Н, OCH_3_), 2.46 (s, 3Н, 4-CH_3_, coincides with the signal of residual protons DMSO-*d*_6_). ^13^C-NMR (100 MHz, DMSO-*d*_6_ + CDCl_3_): δ 161.6 (С=О), 147.7, 138.2, 132.2, 127.4, 127.1, 123.0, 121.3, 118.8, 52.9 (OCH_3_), 17.5 (4-CH_3_). Mass Spectrum (MS) (*m/z*, %): 253 [M]^+^ (4.4), 252 [M − H]^+^ (1.5), 221 [M − СH_3_OH]^+^ (8.4), 195 (80.2), 119 (75.3), 103 (17.0), 93 (100), 92 (59.5), 77 (50.0). Analytically calculated (Anal. Calcd.) for C_11_H_11_NO_4_S: C, 52.16; H, 4.38; N, 5.53; S 12.66%. Found: C, 52.07; H, 4.30; N, 5.46; S 12.72%.

### 2.3. General Procedure for the Synthesis of 1-Alkyl-Substituted Methyl 4-Methyl-2,2-Dioxo-1*H*-2λ^6^,1-Benzothiazine-3-Carboxylates (**5a**–**g**)

To the solution of 2.53 g (0.01 mol) of methyl 4-methyl-2,2-dioxo-1*H*-2λ^6^,1-benzothiazine-3-carboxylate (**3**) in 15 mL of DMSO add 2.07 g (0.015 mol) of K_2_CO_3_ and mix for 30 min. Then add 0.015 mol of the corresponding alkyl halide, and mix within the time specified in Table 1 at 25 °С. Dilute the mixture with cold water and acidify with diluted HCl to рН 4. Extract CH_2_Cl_2_ (3 × 10 mL). Combine the extracts, remove the solvent by distillation (at reduced pressure at the end). Dissolve the residue in 15–20 mL of a hot methanol, purify with activated charcoal and filter. Purify the resulting solution with coal, then place in the freezer (−20 °С) for 24 h. Filter the crystals of methyl 1-alkyl-substituted 4-methyl-2,2-dioxo-1*H*-2λ^6^,1-benzothiazine-3-carboxylate (**5a**–**g**) obtained and dry in the air, and recrystallized from the methanol (esters **5a**–**f**) or acetone (ester **5g**). Esters **5a**–**g** were colorless or white with yellowish crystals.

*Methyl 1-Methyl-4-methyl-2,2-dioxo-1*H*-2λ^6^,1-benzothiazine-3-carboxylate* (**5a**). Yield: 84%; mp 97–98 °C; R*_f_* 0.64; ^1^H-NMR (400 MHz, DMSO-*d*_6_): δ 7.86 (d, 1Н, *J* = 7.1 Hz, Н-5), 7.63 (t, 1Н, *J* = 7.6 Hz, Н-7), 7.42 (d, 1Н, *J* = 8.0 Hz, Н-8), 7.32 (t, 1Н, *J* = 7.6 Hz, Н-6), 3.86 (s, 3Н, OCH_3_), 3.38 (s, 3Н, NCH_3_), 2.47 (s, 3Н, 4-CH_3_, coincides with the signal of residual protons DMSO-*d*_6_). ^13^C-NMR (100 MHz, DMSO-*d*_6_): δ 161.7 (С=О), 146.8, 140.3, 133.1, 128.8, 127.6, 124.4, 122.8, 118.9, 53.7 (OCH_3_), 31.7 (NCH_3_), 17.5 (4-CH_3_). MS (*m/z*, %): 267 [M]^+^ (4.0), 266 [M − H]^+^ (13.8), 235 [M − СH_3_OH]^+^ (6.3), 234 (28.0), 232 (57.0), 201 (100), 187 (90.3), 186 (71.7), 142 (46.1), 141 (48.1), 115 (15.7). Anal. Calcd. for C_12_H_13_NO_4_S: C, 53.92; H, 4.90; N, 5.24; S 12.00%. Found: C, 53.85; H, 4.96; N, 5.31; S 11.93%.

*Methyl 1-Ethyl-4-methyl-2,2-dioxo-1*H*-2λ^6^,1-benzothiazine-3-carboxylate* (**5b**). Yield: 80%; mp 94–95 °C; R*_f_* 0.66; ^1^H-NMR (400 MHz, DMSO-*d*_6_): δ 7.85 (d, 1Н, *J* = 8.4 Hz, Н-5), 7.62 (t, 1Н, *J* = 7.6 Hz, Н-7), 7.52 (d, 1Н, *J* = 8.4 Hz, Н-8), 7.33 (t, 1Н, *J* = 7.2 Hz, Н-6), 3.96 (q, 2H, *J* = 7.0 Hz, NCH_2_), 3.85 (s, 3Н, OCH_3_), 2.46 (s, 3Н, 4-CH_3_, coincides with the signal of residual protons DMSO-*d*_6_), 1.14 (t, 3Н, *J* = 6.8 Hz, NCH_2_CH_3_). ^13^C-NMR (100 MHz, DMSO-*d*_6_): δ 161.4 (С=О), 146.4, 139.2, 132.9, 128.6, 128.4, 124.6, 124.0, 120.2, 53.5 (OCH_3_), 43.2 (NCH_2_), 17.9 (4-CH_3_), 14.3 (NCH_2_CH_3_). MS (*m/z*, %): 281 [M]^+^ (2.7), 280 [M − H]^+^ (10.7), 279 (66.3), 278 (70.5), 277 (100), 276 (67.0), 249 [M − СH_3_OH]^+^ (5.3), 248 (21.7), 246 (27.6), 214 (70.0), 200 (93.9), 199 (99.3), 156 (21.8), 155 (30.3), 141 (45.9), 128 (22.2), 114 (12.1). Anal. Calcd. for C_13_H_15_NO_4_S: C, 55.50; H, 5.37; N, 4.98; S 11.40%. Found: C, 55.57; H, 5.43; N, 5.06; S 11.31%.

*Methyl 1-Allyl-4-methyl-1-propyl-2,2-dioxo-1*H*-2λ^6^,1-benzothiazine-3-carboxylate* (**5c**). Yield: 77%; mp 87–89 °C; R*_f_* 0.71; ^1^H-NMR (400 MHz, DMSO-*d*_6_): δ 7.86 (d, 1Н, *J* = 8.1 Hz, Н-5), 7.60 (t, 1Н, *J* = 7.6 Hz, Н-7), 7.43 (d, 1Н, *J* = 8.1 Hz, Н-8), 7.33 (t, 1Н, *J* = 7.5 Hz, Н-6), 5.89–5.79 (m, 1H, NCH_2_CH), 5.21 (d, 1H, *J* = 16.8 Hz, NCH_2_CH=CH-*trans*), 5.17 (d, 1H, *J* = 10.0 Hz, NCH_2_CH=CH-*cis*), 4.55 (d, 2H, *J* = 4.8 Hz, NCH_2_), 3.85 (s, 3Н, OCH_3_), 2.47 (s, 3Н, 4-CH_3_, coincides with the signal of residual protons DMSO-*d*_6_). ^13^C-NMR (100 MHz, DMSO-*d*_6_): δ 161.2 (С=О), 146.7, 139.5, 133.2, 132.8, 128.6, 128.4, 124.6, 123.7, 120.2, 118.4, 53.6 (OCH_3_), 49.9 (NCH_2_), 17.9 (4-CH_3_). MS (*m/z*, %): 292 [M − H]^+^ (2.5), 291 (11.3), 290 (61.7), 289 (65.4), 288 (86.9), 261 [M − СH_3_OH]^+^ (1.7), 260 (13.8), 258 (22.6), 217 (18.2), 194 (46.9), 168 (36.0), 167 (49.5), 166 (100), 165 (77.3), 164 (70.6), 155 (34.3), 153 (43.8), 141 (20.2), 128 (31.7), 127 (42.7), 114 (10.1), 82 (21.5). Anal. Calcd. for C_14_H_15_NO_4_S: C, 57.32; H, 5.15; N, 4.77; S 10.93%. Found: C, 57.37; H, 5.22; N, 4.85; S 10.85%.

*Methyl 4-Methyl-1-propyl-2,2-dioxo-1*H*-2λ^6^,1-benzothiazine-3-carboxylate* (**5d**). Yield: 75%; mp 82–84 °C; R*_f_* 0.74; ^1^H-NMR (400 MHz, CDCl_3_): δ 7.71 (dd, 1Н, *J* = 7.8 and 1.5 Hz, Н-5), 7.53 (td, 1Н, *J* = 7.8 and 1.5 Hz, Н-7), 7.30 (d, 1Н, *J* = 8.5 Hz, Н-8), 7.26 (td, 1Н, *J* = 7.8 and 1.5 Hz, Н-6), 3.97 (s, 3Н, OMe), 3.93 (t, 2Н, *J* = 7.5 Hz, NCH_2_), 2.57 (s, 3Н, 4-Me), 1.78–1.66 (m, 2Н, NCH_2_CH_2_), 0.90 (t, 3Н, *J* = 7.5 Hz, NCH_2_CH_2_CH_3_). ^13^C-NMR (100 MHz, CDCl_3_): δ 161.4 (С=О), 146.8, 139.5, 132.1, 128.8, 128.4, 124.3, 124.2, 119.3, 53.1 (OCH_3_), 49.2 (NCH_2_), 22.0 (NCH_2_CH_2_), 17.7 (4-CH_3_), 11.1 (NCH_2_CH_2_CH_3_). MS (*m/z*, %): 294 [M − H]^+^ (3.3), 292 (25.8), 291 (32.4), 289 (23.2), 288 (22.6), 263 [M − СH_3_OH]^+^ (3.9), 262 (4.9), 260 (8.5), 219 (31.3), 218 (73.6), 217 (100), 216 (40.1), 156 (16.6), 155 (27.2), 154 (35.4), 128 (20.6), 127 (25.1), 126 (22.4). Anal. Calcd. for C_14_H_17_NO_4_S: C, 56.93; H, 5.80; N, 4.74; S 10.86%. Found: C, 57.01; H, 5.88; N, 4.66; S 10.94%.

*Methyl 1-Isopropyl-4-methyl-2,2-dioxo-1*H*-2λ^6^,1-benzothiazine-3-carboxylate* (**5e**). Yield: 70%; mp 121–123 °C; R*_f_* 0.68; ^1^H-NMR (400 MHz, DMSO-*d*_6_): δ 7.83 (d, 1Н, *J* = 8.0 Hz, Н-5), 7.57 (t, 1Н, *J* = 7.6 Hz, Н-7), 7.50 (d, 1Н, *J* = 8.0 Hz, Н-8), 7.43 (t, 1Н, *J* = 7.2 Hz, Н-6), 4.38–4.31 (m, 1H, NCH), 3.84 (s, 3Н, OCH_3_), 2.42 (s, 3Н, 4-CH_3_), 1.16 (d, 6Н, *J* = 6.4 Hz, NCH_2_(CH_3_)_2_). ^13^C-NMR (100 MHz, DMSO-*d*_6_): δ 161.4 (С=О), 146.0, 138.3, 132.0, 131.0, 128.1, 127.5, 126.8, 125.9, 56.0 (NCH), 53.4 (OCH_3_), 21.6 (NCH(CH_3_)_2_), 17.7 (4-CH_3_). MS (*m/z*, %): 294 [M − H]^+^ (2.4), 292 (21.4), 291 (28.1), 289 (26.1), 288 (18.7), 263 [M − СH_3_OH]^+^ (1.6), 262 (4.4), 260 (7.3), 219 (45.7), 218 (100), 217 (84.4), 216 (42.3), 156 (14.5), 155 (21.0), 154 (31.5), 128 (22.8), 127 (33.0), 126 (19.7). Anal. Calcd. for C_14_H_17_NO_4_S: C, 56.93; H, 5.80; N, 4.74; S 10.86%. Found: C, 57.00; H, 5.86; N, 4.69; S 10.91%.

*Methyl 1-Butyl-4-methyl-2,2-dioxo-1*H*-2λ^6^,1-benzothiazine-3-carboxylate* (**5f**). Yield: 73%; mp 87–88 °C; R*_f_* 0.77; ^1^H-NMR (400 MHz, DMSO-*d*_6_): δ 7.85 (d, 1Н, *J* = 8.0 Hz, Н-5), 7.61 (t, 1Н, *J* = 7.6 Hz, Н-7), 7.51 (d, 1Н, *J* = 8.4 Hz, Н-8), 7.33 (t, 1Н, *J* = 7.6 Hz, Н-6), 3.93 (t, 2Н, *J* = 7.4 Hz, NCH_2_), 3.85 (s, 3Н, OCH_3_), 2.44 (s, 3Н, 4-CH_3_), 1.51–1.47 (m, 2Н, NCH_2_CH_2_), 1.19–1.16 (m, 2Н, NCH_2_CH_2_CH_2_), 0.80 (t, 3Н, *J* = 7.4 Hz, NCH_2_CH_2_CH_2_CH_3_). ^13^C-NMR (100 MHz, DMSO-*d*_6_): δ 161.4 (С=О), 145.9, 139.3, 132.8, 128.6, 128.4, 124.5, 124.0, 120.1, 53.5 (OCH_3_), 46.8 (NCH_2_), 30.3 (NCH_2_CH_2_), 19.6 (NCH_2_CH_2_CH_2_), 17.9 (4-CH_3_), 13.7 (NCH_2_CH_2_CH_2_CH_3_). MS (*m/z*, %): 308 [M − H]^+^ (2.7), 306 (20.2), 305 (25.7), 304 (27.2), 303 (16.0), 277 [M − СH_3_OH]^+^ (1.8), 276 (11.3), 274 (12.5), 233 (41.8), 232 (82.1), 231 (100), 230 (40.5), 128 (20.1), 127 (27.2), 126 (14.3). Anal. Calcd. for C_15_H_19_NO_4_S: C, 58.23; H, 6.19; N, 4.53; S 10.36%. Found: C, 58.15; H, 6.13; N, 4.58; S 10.43%.

*Methyl 1-Benzyl-4-methyl-2,2-dioxo-1*H*-2λ^6^,1-benzothiazine-3-carboxylate* (**5g**). Yield: 86%; mp 113–115 °C; R*_f_* 0.78; ^1^H-NMR (400 MHz, DMSO-*d*_6_): δ 7.81 (d, 1Н, *J* = 8.0 Hz, Н-5), 7.52 (t, 1Н, *J* = 7.6 Hz, Н-7), 7.41 (d, 1Н, *J* = 8.0 Hz, Н-8), 7.33 (t, 1Н, *J* = 7.2 Hz, Н-6), 7.30–7.18 (m, 5H, Ph), 5.19 (s, 2H, NCH_2_), 3.88 (s, 3Н, OCH_3_), 2.42 (s, 3Н, 4-CH_3_). ^13^C-NMR (100 MHz, DMSO-*d*_6_): δ 161.3 (С=О), 146.7, 139.1, 136.2, 132.8, 129.2, 128.9, 128.6, 128.5, 128.3, 128.0, 127.5, 124.7, 124.0, 120.3, 53.6 (OCH_3_), 50.1 (NCH_2_), 17.9 (4-CH_3_). MS (*m/z*, %): 342 [M − H]^+^ (2.3), 340 (12.4), 339 (18.9), 338 (11.5), 310 [M − H − СH_3_OH]^+^ (3.7), 218 (11.7), 217 (11.8), 178 (16.5), 90 (83.0), 89 (100). Anal. Calcd. for C_18_H_17_NO_4_S: C, 62.96; H, 4.99; N, 4.08; S 9.34%. Found: C, 63.04; H, 5.06; N, 4.00; S 9.42%.

*Methyl 1-Benzyl-4-methyl-2,2-dioxo-1*H*-2λ^6^,1-benzothiazine-3-carboxylate Methanol Monosolvate* (**6**). It was obtained by conventional crystallization of *N-*benzyl substituted ester **5g** from methanol. Yield: 94%; mp 104–106 °C (a double-end sealed capillary).

### 2.4. X-ray Structural Analysis of Methyl 1-Isopropyl-4-Methyl-2,2-Dioxo-1*H*-2λ^6^,1-Benzothiazine-3-Carboxylate (**5e**)

The crystals of ester **5e** (C_14_H_17_NO_4_S) were monoclinic, colorless. At 20 °C: *a* 7.5676(3), *b* 15.1539(5), *c* 12.9436(5) Å; β 94.171(4)°; *V* 1480.4(1) Å^3^, *Z* 4, space group Р2_1_/n, *d*_calc_ 1.446 g/cm^3^, µ(MoK_α_) 0.237 mm^−1^, *F*(000) 684. The unit cell parameters and intensities of 15,471 reflections (4323 independent reflections, *R*_int_ = 0.044) were measured on an Xcalibur-3 diffractometer (Oxford Diffraction Limited, Oxford, UK) using MoK_α_ radiation, a Charge Coupled Device (CCD) detector, graphite monochromator, and ω-scanning to 2θ_max_ 60°. The structure was solved by the direct method using the SHELXTL program package (Institute of Inorganic Chemistry, Göttingen, Germany) [25]. The restrictions on the bond lengths were applied for the disordered isopropyl (1.54 Å). The positions of the hydrogen atoms were found from the electron density difference map and refined using the “rider” model with *U*_iso_ = *nU*_eq_ for the non-hydrogen atom bonded to a given hydrogen atom (*n* = 1.5 for methyl, and *n* = 1.2 for the other hydrogen atoms). The structure was refined using *F*^2^ full-matrix least-squares analysis in the anisotropic approximation for non-hydrogen atoms to *wR*_2_ 0.229 for 4,218 reflections (*R*_1_ 0.072 for 2386 reflections with *F* > 4σ (*F*), *S* 1.035). CCDC 1518396 contains the supplementary crystallographic data for this paper. These data can be obtained free of charge from the Cambridge Crystallographic Data Center [26].

### 2.5. X-ray Structural Analysis of Methyl 1-Benzyl-4-Methyl-2,2-Dioxo-1*H*-2λ^6^,1-Benzothiazine-3-Carboxylate Methanol Monosolvate (**6**)

The crystals of *N-*benzyl substituted ester methanol monosolvate **6** (C_1__8_H_1__7_NO_4_S · СН_3_ОH) were monoclinic, colorless. At 20 °C: *a* 8.4692(5), *b* 14.835(1), *c* 16.334(1) Å; β 100.271(6)°; *V* 2019.4(2) Å^3^, *Z* 4, space group Р2_1_/n, *d*_calc_ 1.235 g/cm^3^, µ(MoK_α_) 0.187 mm^−1^, *F*(000) 792. The unit cell parameters and intensities of 21,575 reflections (5865 independent reflections, *R*_int_ = 0.084) were measured on an Xcalibur-3 diffractometer (Oxford Diffraction Limited) using MoK_α_ radiation, a CCD detector, graphite monochromator, and ω-scanning to 2θ_max_ 60°. The structure was solved by the direct method using the SHELXTL program package (Institute of Inorganic Chemistry) [25]. The restrictions on the bond lengths were applied for the disordered methanol molecule (1.43 Å). The positions of the hydrogen atoms were found from the electron density difference map and refined using the “rider” model with *U*_iso_ = *nU*_eq_ for the nonhydrogen atom bonded to a given hydrogen atom (*n* = 1.5 for methyl and hydroxyl groups, and *n* = 1.2 for the other hydrogen atoms). The structure was refined using *F*^2^ full-matrix least-squares analysis in the anisotropic approximation for non-hydrogen atoms to *wR*_2_ 0.242 for 5,762 reflections (*R*_1_ 0.081 for 2351 reflections with *F* > 4σ (*F*), *S* 0.874). CCDC 1518395 contains the supplementary crystallographic data for this paper. These data can be obtained free of charge from the Cambridge Crystallographic Data Center [27].

### 2.6. Pharmacology

#### Analgesic Test

The analgesic activity of the synthesized methyl 4-methyl-2,2-dioxo-1*H*-2λ^6^,1-benzothiazine-3-carboxylates (**3**) and its *N*-alkyl-substituted analogues **5a**–**g** were studied compared to piroxicam (Jenapharm, Jena, Germany) and meloxicam (Boehringer Ingelheim, Ingelheim am Rhein, Germany) being similar to the structure on the model of the thermal tail-flick procedure in white male rats weighing 180–200 g (Tail Immersion Test) [28]. For this purpose, the rat’s tail tip was immersed in a water bath heated to 54 °С, and the latent period of the tail withdrawal (immersion) expressed in seconds was determined. The analgesic effect (in %) was assessed by the change of the latent period 2 h after introduction of the test substances and the reference drugs. Seven experimental animals were involved to obtain statistically reliable results (the significance level of the confidence interval accepted in this work was *p* ≤ 0.05) in testing each of esters **3** and **5a**–**g**, the reference drugs and control. All substances under research, piroxicam, and meloxicam were introduced orally in the form of fine aqueous suspensions stabilized with Tween-80 in a screening dose of 20 mg/kg. The animals of the control group received an equivalent amount of water with Tween-80. 

All biological experiments were carried out in full accord with the European Convention on the Protection of Vertebrate Animals Used for Experimental and Other Scientific Purposes and the Ukrainian Law No. 3447-IV “On protection of animals from severe treatment” (2006) (project ID 3410U14 approved October 15, 2015).

## 3. Results and Discussion

### 3.1. Chemistry

**Scheme 2:**
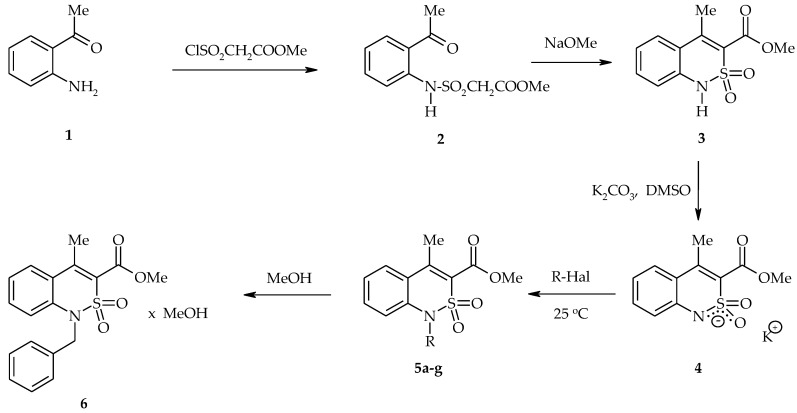
New synthesis of *N*-alkyl substituted methyl 4-methyl-2,2-dioxo-1*H*-2λ^6^,1-benzothiazine-3-carboxylates **5a**–**g**. **5**: (**а**) R = –CH_3_; (**b**) R = –C_2_H_5_; (**c**) R = –CH_2_CH=CH_2_; (**d**) R = –C_3_H_7_; (**e**) R = –CH(CH_3_)_2_; (**f**) R = –C_4_H_9_; (**g**) R = –CH_2_-C_6_H_5_.

The initial methyl 4-methyl-2,2-dioxo-1*H*-2λ^6^,1-benzothiazine-3-carboxylate (**3**) was obtained by the traditional linear scheme with a good yield and purity. This synthesis does not require special explanation. It should be only noted that treatment of type **2** anilides with alkali metal alcoholates is often accompanied by complete or partial transesterification [10]. Therefore, as a basic catalyst of heterocyclization it is necessary to use the alcoholate prepared from the same alcohol, which fragment is already contained in alkyl (chlorosulfonyl)acetate: it is clear that in our case it is sodium methylate (Scheme 2). When meeting this simple condition the target methyl ester **3** is formed, but not its mixtures that are difficult to separate with other 3-alkoxycarbonyl analogues in various ratios.

In the interaction of alkyl halides and salts that are structurally close to methyl 4-hydroxy-2,2-dioxo-1*H*-2λ^6^,1-benzothiazine-3-carboxylates, as it is known, the isomeric products of 3-C-alkylation are formed rather easily and with high yields in addition to 4-*О*-alkyl-substituted derivatives being typical for such reactions [29]. The similar electrophilic attack of position 3 in the anion of the potassium salt of methyl 4-methyl-2,2-dioxo-1*H*-2λ^6^,1-benzothiazine-3-carboxylate (**4**) is unlikely. However, at least theoretically, a possibility of occurrence of other side chemical processes in the course of the reaction studied is not excluded. Consequently, one of the key aspects of our study is confirmation of the purity of the resulting compounds and unambiguous identification of their structure. 

A series of experiments conducted with TLC-monitoring has shown that the reaction of the potassium salt anion of methyl 4-methyl-2,2-dioxo-1*H*-2λ^6^,1-benzothiazine-3-carboxylate (**4**) and alkyl halides proceeds rather quickly already at room temperature in the DMSO/K_2_CO_3_ system. For example, in the experiment with a highly reactive allyl bromide the initial ester **3** is not chromatographically detectable in the reaction mixture already in 0.5 h after the start of alkylation. With the less reactive alkyl halides the reaction duration naturally increases, but it must not significantly exceed the time limits experimentally found and recommended by us (Table 1). The cause for this restriction is simple—as it turned out, the initially formed alkyl-substituted esters **5a**–**g** do not differ in chemical stability, and according to TLC-monitoring they gradually undergo further deeper structural changes under the synthetic conditions, therefore, these esters require a separate and more detailed study.

**Figure 2:**
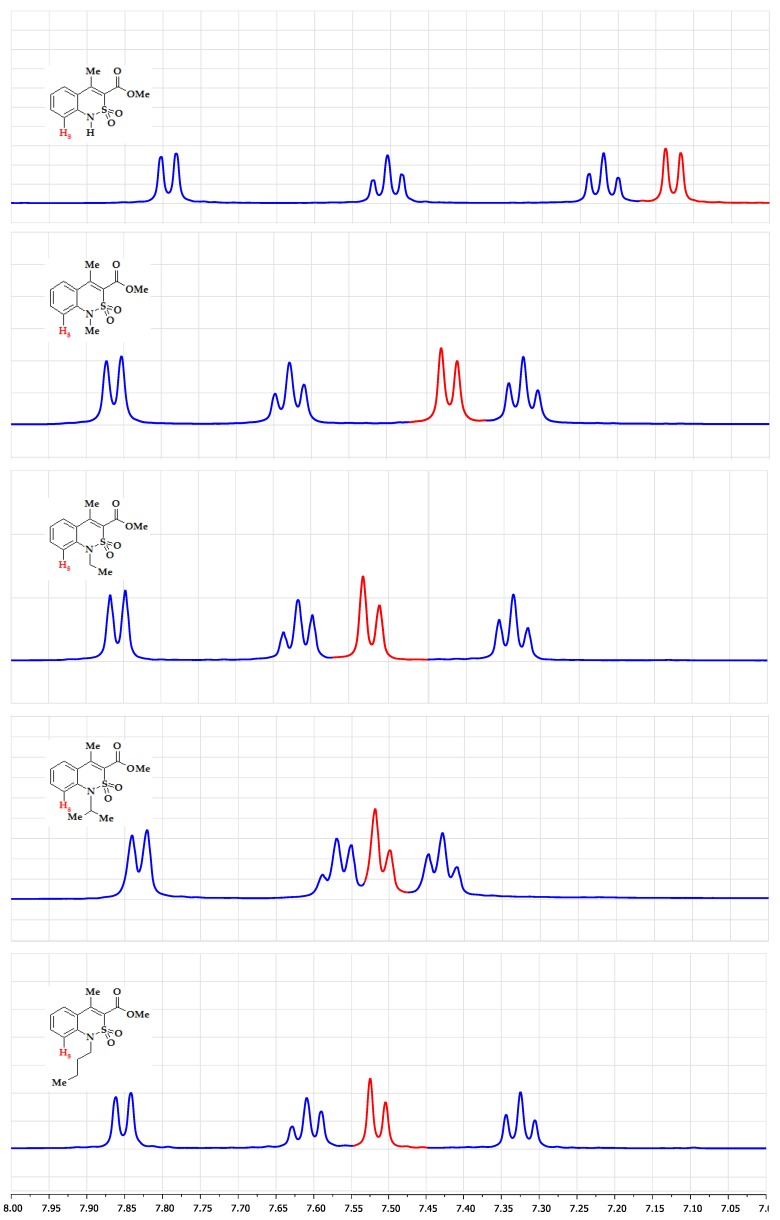
Fragments of ^1^H-NMR spectra (the signals of aromatic protons) of ester **3** and some of its *N*-alkyl-substituted derivatives **5**. The signal of proton Н-8 is marked in red color.

^1^Н- and ^13^C-NMR spectra of all esters of benzothiazine-3-carboxylic acids **3** and **5** synthesized fully comply with the structure attributed to them (see Materials and Methods). Essentially, a simple comparison of the spectral data obtained allows to solve the main structural analytical task of this study—to determine the true direction of alkylation—even without the use of special methods of NMR spectroscopy. In particular, the chemical shifts of alkyl carbon atoms in ^13^C-NMR spectra suggest that, in general, the substituent is either at the atom of nitrogen or oxygen (the last variant is possible as a result of the exchange of alkyl groups in the ester fragment). A comparative analysis of the “aromatic” region of the ^1^H-NMR spectra of the starting ester **3** and its alkyl-substituted derivatives **5a**–**g** allows excluding the oxygen atom of the potential reaction centers. The basis for this conclusion was the fact that after introduction of the alkyl substituent the spectral pattern changes significantly (Figure 2). The resonance signals of all aromatic protons undergo a paramagnetic shift, but in this respect, the signal of Н-8 proton is particularly illustrative, it shifts downfield by 0.3 ppm in the spectrum of methyl-substituted ester **5a** compared to unalkylated analog **3**. With the increase in size of the alkyl substituent the shift of the specified signal also increases—it reaches 0.4 ppm in ethyl derivative **5b**. Subsequently the signal of Н-8 proton remains approximately in the same position irrespective of the spatial structure of the alkyl substituent. Even the isopropyl fragment of ester **5e** does not change the situation. However, this substituent being the most volumetric sterically brings its own corrections, it noticeably shifts downfield the signals not only the neighboring H-8, but also protons, which are more remote in space, in particular Н-6 proton (Figure 2). In our opinion, the strong interaction observed in the experimental ^1^H-NMR spectra between Н-8 proton and alkyl substituents introduced is a typical manifestation of the van der Waals effect [30]. Consequently, these two fragments are close together in space.

Mass spectrometry also gives important and useful information about the structure of the compounds synthesized. For example, in the case of the lower homologs—esters **3** and **5a**,**b**—the molecular mass of each test sample can be determined. Other esters do not possess stability in the conditions of electron impact ionization, which is sufficient for formation of molecular cation-radicals, but there are peaks that are close to the mass of fragmented ions [M − H]^+^ in their mass spectra. The common feature, which characterizes the mass spectrometric behavior of all the compounds studied, is release of the methanol molecule from their molecular ions. This is evidenced by the appearance of peaks of the corresponding fragmentation ions [M − СH_3_OH]^+^ or [M − H − СH_3_OH]^+^ in the mass spectra.

**Figure 3:**
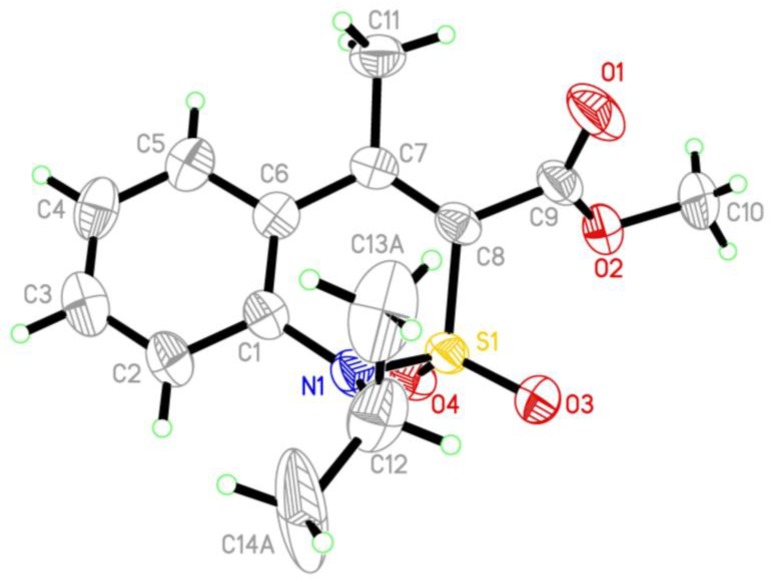
The molecular structure of *N-*isopropyl-substituted ester (**5e**) with atoms represented by thermal vibration ellipsoids of 50% probability.

**Figure 4:**
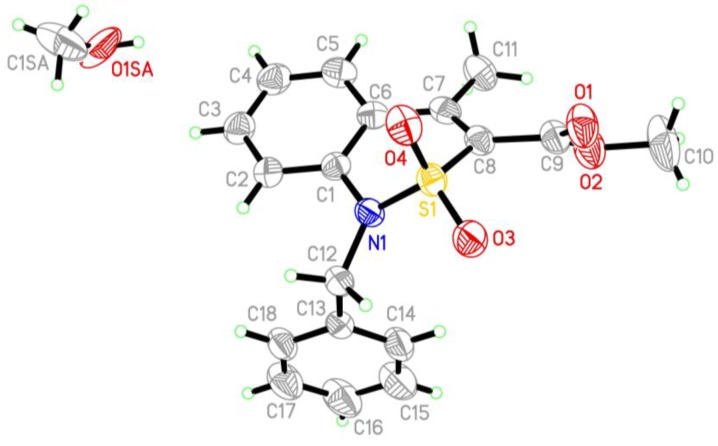
The molecular structure of *N-*benzyl-substituted ester methanol monosolvate (**6**) with atoms represented by thermal vibration ellipsoids of 50% probability.

Naturally, X-ray diffraction analysis gives the most complete picture of the real structure of alkyl-substituted esters **5a**–**g** obtained. By the example of allyl [31], isopropyl (Figure 3) and benzyl (Figure 4) derivatives it has been conclusively proven that actually methyl 4-methyl-2,2-dioxo-1*H*-2λ^6^,1-benzothiazine-3-carboxylate (**3**) is alkylated exclusively at the nitrogen atom independently from the structure of alkyl halide. Simultaneously, it has been found that the tendency to form solvates with various organic solvents previously mentioned for 4-hydroxy-2,2-dioxo-1*H*-2λ^6^,1-benzothiazines over and over again [17,32] is also typical for their 4-methyl-substituted analogs. In particular, the ability of methyl 1-benzyl-4-methyl-2,2-dioxo-1*H*-2λ^6^,1-benzothiazine-3-carboxylate (**5g**) to form a monosolvate easily from methanol already in crystallization has been found (Figure 4). 

The features of the spatial structure of 1-alkyl-substituted methyl 4-methyl-2,2-dioxo-1*H*-2λ^6^,1-benzothiazine-3-carboxylates are briefly as follows. The dihydrothiazine cycle adopts a distorted sofa conformation in these compounds (the puckering parameters [33] are: S = 0.68, Θ = 55.5°, Ψ = 28.7° for ester **5e** and S = 0.68, Θ = 54.6°, Ψ = 24.8° for ester **5g**). Deviations of the S_(1)_ and C_(8)_ atoms from the mean plane of the remaining atoms of the corresponding cycles are 0.99 Å and 0.39 Å for compound **5e** and 0.97 Å and 0.34 Å for compound **5g**, respectively. The N_(1)_ atom has almost planar configuration where the sum of bond angles centered at it is 355° for **5e** and 357° for **5g**. 

The strong steric repulsion between atoms of the methyl group at the C_(7)_ atom and atoms of the aromatic ring (the shortened intramolecular contacts are: Н_(5)_…С_(11)_ 2.56 Å in **5e** and 2.59 Å in **5g** (the van der Waals radii sum [34] is 2.87 Å), Н_(11)_…С_(5)_ 2.83 Å in **5e** and 2.86 Å in **5g** (2.87 Å), Н_(5)_…Н_(11)_ 2.28 Å in **5e** and 2.31 Å in **5g** (2.34 Å)) results in the disturbance of the conjugation between aromatic ring and C_(7)_–C_(8)_ double bond π-systems (the С_(5)_–С_(6)_–С_(7)_–С_(8)_ torsion angle is −161.2(3)° in **5e** and 161.8(3)° in **5g**). The ester substituent is turned significantly to the S_(1)_–С_(8)_ endocyclic bond (the S_(1)_–С_(8)_–С_(9)_–О_(1)_ torsion angle is −137.1(3)° in **5e** and −36.3(4)° in **5g**). At this the С_(11)_–Н_(11__а)_…О_(1)_ (Н…О 2.31 Å, С–Н…О 132°) intramolecular hydrogen bond and С_(11)_–Н_(11__а)_…О_(2)_ (Н…О 2.22 Å, С–Н…О 132°) hydrogen bond have appeared in **5e** and **5g**, respectively. The С_(7)_–С_(8)_ double bond is slightly elongated (1.358(4) Å in **5e** and 1.370(4) Å in **5g** as compared to its mean value [35] 1.326 Å) due to conjugation with π-systems of neighboring fragments. The C_(1)_–N_(1)_ bond is elongated up to 1.434(3) Å in **5e** and 1.414(4) Å in **5g** (its mean value is 1.371 Å).

### 3.2. Evaluation of the Analgesic Activity

The pharmacological tests conducted have fully confirmed the expediency of our studies undertaken and shown that the transfer from methyl 1-R-4-hydroxy-2,2-dioxo-1*H*-2λ^6^,1-benzothiazine-3-carboxylates **I** to their 4-methyl-substituted analogs **II** presented in Figure 1 is really bioisosteric. The proof of this is the fact that all methyl 1-R-4-methyl-2,2-dioxo-1*H*-2λ^6^,1-benzothiazine-3-carboxylates **3** and **5a**–**g** synthesized showed pronounced analgesic properties (Table 2). Moreover, by the ability to inhibit the thermal pain response of the animals tested most of them are not inferior and even significantly superior to piroxicam, which was the first in the group of oxicams—new non-steroidal anti-inflammatory drugs with a good analgesic effect, and to date has become a classic standard for biological research.

Of all methyl 1-R-4-methyl-2,2-dioxo-1*H*-2λ^6^,1-benzothiazine-3-carboxylates presented in this work *N*-methyl derivative **5a** deserves special attention. As an oral analgesic this compound was much more effective of not only piroxicam, but its modern and a more active analogue—meloxicam. All this allows recommending ester **5а** for extensive pharmacological testing on other experimental models. In addition, our results can be regarded somewhat broader, for example, as the substantiation for involving not only esters described in the compounds under study, but 1-R-4-methyl-2,2-dioxo-1*H*-2λ^6^,1-benzothiazine-3-carboxylic acids as their basis, as well as various *N*-R-amides. Moreover, *N*-R-amides were specified as the most promising analgesics in the series of 4-hydroxy-2,1-benzothiazine analogs previously studied [12,13,14,15,16,17,18,19].

## 4. Conclusions

The present study describes methyl 1-R-4-methyl-2,2-dioxo-1*H*-2λ^6^,1-benzothiazine-3-carboxylates synthesized as potential bioisosteres of analgesics from a series of 4-hydroxy-2,1-benzothiazines. The complex use of X-ray diffraction analysis, NMR (^1^Н and ^13^С) spectroscopy and mass spectrometry has allowed to prove convincingly that methyl 4-methyl-2,2-dioxo-1*H*-2λ^6^,1-benzothiazine-3-carboxylate is alkylated by alkyl halides on the nitrogen atom in the DMSO/K_2_CO_3_ system. Simultaneously on the example of *N-*benzyl derivative the ability of 4-methyl-2,2-dioxo-2,1-benzothiazines to form solvates with methanol has been found, and it is useful to remember when working with substances of this chemical class. The pharmacological screening has revealed highly active analgesics being of interest for more detailed study among the compounds synthesized.

## Figures and Tables

**Table 1:** Alkylation of Methyl 4-Methyl-2,2-dioxo-1*H*-2λ^6^,1-benzothiazine-3-carboxylate (**3**), 25 °C.

Entry	Product	Alkyl Halide	Duration of the Reaction (h)
1	5a	CH_3_–I	1.3
2	5b	C_2_H_5_–I	2.0
3	5c	H_2_C=CH–CH_2_–Br	0.5
4	5d	C_3_H_7_–I	2.4
5	5e	(CH_3_)_2_CH–I	5.0
6	5f	C_4_H_9_–I	2.5
7	5g	C_6_H_5_–CH_2_–Cl ^1^	1.5

^1^ In alkylation 0.5 g of NaI is added in the reaction mixture.

**Table 2:**
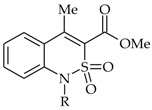
The Analgesic Activity of Esters **3** and **5a**–**g**, and Reference Drugs.

Entry	Product	R	Latent Period in 2 h after Introduction of the Compounds (s) ^1^	Change of the Latent Period, Compared to Control (%) ^2^
1	**3**	H	8.47 ± 0.38	+17.6
2	**5a**	–CH_3_	12.51 ± 1.04	+73.7 (+10.9)
3	**5b**	–C_2_H_5_	10.29 ± 0.61	+42.9 (+23.6)
4	**5c**	–CH_2_–CH=CH_2_	9.56 ± 0.44	+32.8 (+71.1)
5	**5d**	–C_3_H_7_	8.22 ± 0.35	+14.2
6	**5e**	–CH(CH_3_)_2_	9.21 ± 0.43	+27.9
7	**5f**	–C_4_H_9_	8.60 ± 0.39	+19.5
8	**5g**	–CH_2_–C_6_H_5_	9.85 ± 0.46	+36.8
9	Meloxicam	–	11.59 ± 1.02	+61.1
10	Piroxicam	–	9.24 ± 0.45	+28.4
11	Control	–	7.20 ± 0.31	–

^1^ All results from biological tests were analyzed statistically using Student’s *t*-test. Effects were regarded as statistically significant at *p ≤* 0.05; ^2^ The data on the analgesic activity of the corresponding methyl 4-hydroxy-1-R-2,2-dioxo-1*H*-2λ^6^,1-benzothiazine-3-carboxylates [10] are given in parentheses.
